# Role of Renal Resistive Index as an Early Marker of Diabetic Nephropathy in Children With Type 1 Diabetes Mellitus

**DOI:** 10.7759/cureus.82202

**Published:** 2025-04-13

**Authors:** Preeti Choudhary, Meenakashi Rani, G S Sengar

**Affiliations:** 1 Pediatrics, Sardar Patel Medical College, Bikaner, IND

**Keywords:** diabetic nephropathy, pediatric diabetic ketoacidosis, renal resistive index, type 1 diabetes mellitus, urine albumin excretion

## Abstract

Objective

Diabetic nephropathy (DN) is the most common microvascular complication in type 1 diabetes mellitus (T1DM). The study aimed to assess the role of renal resistive index (RRI) in early detection of DN in children with T1DM.

Methods

This study was conducted on 122 children with T1DM. The following parameters were studied: age, gender of patients, duration of diabetes, number of diabetic ketoacidosis (DKA) episodes, serum creatinine, serum urea, urine albumin excretion (UAE), glycated hemoglobin (HBA1c) and mean RRI of both kidneys.

Results

The study included 60 (49%) males and 62 (51%) females; with male to female ratio 0.96:1; their mean ages were 9.5 ± 2.89 years (range, 5-14) years and mean disease duration was 3.7 ± 1.6 years (range, 2-10) years; mean value of HBA1c was 11.69 ± 2.1 and 88.5% (108) of cases with T1DM in our study were normoalbuminuric and only 11.4% (14) of cases had albuminuria. The RRI >=0.7 (indicative of DN) was found in 12.2% (15) cases of T1DM in our study. Risk factors significantly associated with DN were age of children (older ages more affected), longer duration of disease, and higher total cholesterol and triglycerides levels. The cases of T1DM with UAE >30 mg/24 hours as well as RRI >=0.7 had significantly higher mean blood urea and serum creatinine levels indicating renal involvement.

Conclusion

RRI values significantly correlates with renal UAE. RRI abnormality occurs even before the level of UAE reaches the cut-off value of early diagnosis of DN. Hence, RRI (>=0.7) can be used as an early indicator of DN.

## Introduction

Type 1 diabetes mellitus (T1DM) leads to an increased risk of morbidity and early mortality due to chronic complication affecting both the micro- and macro-vasculature. In T1DM, microvascular complications commonly include nephropathy, retinopathy, and neuropathy. However, these complications may also impact cognitive function, the heart, and other organs. The main risk factor for microvascular disease is hyperglycaemia, and reducing glycated hemoglobin (HbA1C) through intensive diabetes management, particularly early during the disease, is associated with striking reductions in incidence and slower progression of microvascular disease. Macrovascular complications of T1DM manifest mainly as atherosclerosis and thrombosis in the heart, peripheral arteries, and brain. Diabetic nephropathy (DN), whether presenting as microalbuminuria, macroalbuminuria, or a decline in glomerular filtration rate, progressively increases the risk of macrovascular complications [1].

Over the past 25 years, the incidence of microvascular and macrovascular complications in individuals with T1DM has significantly declined, leading to improved outcomes [2,3]. The enhancement in outcomes is primarily attributed to improved glycemic control and more effective management of related risk factors, such as hypertension and hyperlipidemia.

DN is a significant chronic complication of T1DM, impacting around 20-30% of individuals and increasing the risk of cardiovascular disease and end-stage renal disease (ESRD) [4,5]. DN is the leading cause of mortality in T1DM [6-8]. Recognized as the earliest clinical indicator of DN, microalbuminuria occurs in roughly 50% of individuals with T1DM over their lifetime and advances at an annual rate of about 2-3% [9]. In patients with T1DM, an early decline in renal function is commonly observed, especially among those with microalbuminuria [10]. Metabolic and hemodynamic factors contribute to the development of DN, probably by interacting with a genetic susceptibility [11,12].

Diabetic kidney disease (DKD) is traditionally diagnosed based on estimated glomerular filtration rate (eGFR) and albuminuria, defined as a persistent urinary albumin-to-creatinine ratio of ≥30 mg/g and/or a sustained eGFR decline below 60 ml/min per 1.73 m². Clinical factors such as diabetes duration and the presence of diabetic retinopathy also contribute to the diagnosis [13-15]. Annual screening for DKD is recommended for individuals with T1DM, starting five years after diagnosis. In patients with albuminuria, if diabetic retinopathy is present it is strongly suggestive of DKD. DN typically progresses through a prolonged asymptomatic phase before any clinical signs or symptoms appear. There is an urgent need for improved methods to detect early indicators of renal damage to prevent both the onset and advancement of DN.

Renal resistive index (RRI) calculated by using renal Doppler can be a valuable tool in detecting functional alterations in renal hemodynamic. Renal resistivity index is a measure of the hemodynamic changes in the renal arteries. In DN, alterations in vascular valve compliance and resistance influence the resistivity index. Early blood flow changes can be detected using renal Doppler, providing insight into the progression of DN.

## Materials and methods

This is a hospital-based cross-sectional observational study conducted on 122 patients aged five to 15 years having T1DM for at least two years duration. The study was conducted in the Department of Paediatrics, associated group of hospitals attached to S.P. Medical College, Bikaner, during the period from January 2022 to December 2022. The study was conducted after securing approval from the institutional ethical committee. Written consent was obtained from the patients' parents. Those who declined participation were excluded.

Sample size 

The prevalence of DN complications as reported in previous studies varies from 40.00% to 50.00% [4]. We have taken the average prevalence of 45.00% for sample size calculation (n=4pq/d2). Taking allowable error (20.00%) of the reported prevalence of 45%, the sample size is calculated to be 120.

Exclusion criteria 

Children younger than five years and older than 15 years were excluded from the study. Individuals with a documented history of renal artery stenosis, defined as a ≥50% reduction in the luminal diameter of the renal artery, were also excluded. Blood pressure measurements were taken into account, and any participants diagnosed with hypertension were not eligible for inclusion. Furthermore, patients presenting with hydronephrosis in conjunction with clinical signs or symptoms of a urinary tract infection were excluded from participation.

Inclusion criteria 

Children between five and 15 years of age, diagnosed with type 1 diabetes mellitus for a minimum duration of two years, were included in the study after their parental consent.

Patients presenting to paediatrics hospital who fulfilled the inclusion and exclusion criteria were enrolled for study. A comprehensive history taking, physical examination and lab investigations were carried out and data was collected as follows below.

In history, patient’s parents were asked about their education status, demographic characteristics, the year in which the child was diagnosed and number of hospitalization admissions due to DKA episodes since diagnosis. History of symptoms suggesting any microvascular or macrovascular complication was taken. The duration of insulin regimen, compliance with therapy and glycaemic status were also recorded.

A complete general physical and systemic examination was done which included weight, height and BMI. Heart rate, respiratory rate and blood pressure were recorded.

Lab investigations were conducted which included random blood glucose, CBC, lipid profile, renal function test, HbA1c level, and urine examination, including urine microalbumin level. For urine microalbumin level 24-hour urine specimen was collected and measured using microalbumin-turbilatex test (a quantitative turbidimetric test) done on an auto-analyser (Beckman Coulter AU680; Brea, CA, USA). According to this test, microalbuminuria is present if the excretion rate of albumin is between 20 and 200 mg/L (or 30 to 300 mg/24 hours). Subjects with urine albumin excretion (UAE) < 30 mg/24 hours were defined as having normoalbuminuria.

All participants underwent duplex Doppler ultrasonography, performed by a single experienced operator with over 10 years of expertise, ensuring consistency and minimizing inter-observer variability. Imaging was conducted using a Mindray Color Doppler Ultrasound machine (DC-30 model; Shenzhen, China) in both real-time one-color-coded Doppler and pulse Doppler modes. The ultrasound probe was positioned gently on the flank in oblique projection, and the kidney was visualized as a longitudinal image. RRI in our study was calculated in renal segmental arteries bilaterally. RRI was calculated by the built-in software as follows: RRI = peak systolic velocity-end-diastolic velocity/peak systolic velocity. For the study, the mean RRI of both kidneys was used for analysis.

Data statistical analysis

Data were coded, tabulated, and analyzed using the Statistical Package for the Social Sciences (SPSS), version 22.0 (IBM Corp., Armonk, NY, USA).

Descriptive statisticswere used to summarize the data: numerical parametric data were expressed as mean ± standard deviation (SD), along with minimum and maximum values, while categorical variables were presented as frequencies and percentages.

For analytical statistics, the Chi-square test was used to compare qualitative variables between two groups. The independent t-test was applied to compare quantitative data with a parametric distribution between two independent groups, and one-way Analysis of Variance (ANOVA) was used for comparing more than two independent groups with parametric data. Spearman’s correlation coefficient was used to assess the relationship between two quantitative variables within the same group.

Receiver Operating Characteristic (ROC) curveanalysis was conducted to determine the optimal cut-off point, evaluating sensitivity, specificity, positive predictive value (PPV), and negative predictive value (NPV).

A 95% confidence interval was used, and the accepted margin of error was set at 5%. Statistical significance was considered at P < 0.05, while results were regarded as highly significant at P < 0.01 and not significant at P > 0.05.

## Results

This hospital based cross sectional observational study was conducted on 122 patients aged five to 15 years having T1DM for at least two years duration (Table 1). Mean age of cases was 9.5 ± 2.89 years with the male-to-female ratio being 0.96:1.

**Table 1:** Distribution of cases according to age

Age distribution	Number of patients (n, %)
5-9 years	56 (45.9 %)
>9-12 years	43 (35.2 %)
>12-15 years	23 (18.8 %)
Total	122 (100 %)

The majority of cases (90 patients, 73.7%) were having the disease for two to four years (Table 2). The mean duration of disease was 3.7 ± 1.6 years.

**Table 2:** Distribution of cases according to duration of type I diabetes

Duration of diabetes	Number of patients (n, %)
2-4 years	90 (73.7 %)
>4 -6 years	22 (18.0 %)
>6 years	10 (8.1 %)
Total	122 (100 %)

Three or more episodes of DKA occurred in 59 (48.3 %) of studied cases. The mean value of HbA1c was 11.69 ± 2.1. Albuminuria (>30 mg/24 hours) was present in 14 (11.4%) cases of T1DM (Table 3).

**Table 3:** Distribution of cases according to Urine Albumin Excretion level

Parameter	Number of patients (n, %)
Normoalbuminuric (<=30 mg/24 hrs.)	108 (88.5 %)
Albuminuria (> 30 mg/24 hrs.)	14 (11.4 %)
Total	122 (100 %)

Though a trend of increase in UAE was observed with increasing age, it was not statistically significant. The number of cases with UAE (>30 mg/24 hours) was proportionately more in patients belonging to the higher age group but the difference was not statistically significant (Table 4).

**Table 4:** Urine albumin excretion in relation to age of the Patients *Chi-square test Chi-square score- 2.0871

Age of patients (years)	Number of cases (n)	Number of cases (n, %) with Urine albumin excretion (<=30 mg/24 hrs.)	Number of cases (n, %) with Urine albumin excretion (>30 mg/24 hrs.)	p-value*
5-9	56	52 (92.8 %)	4 (7.1 %)	0.35
>9-12	43	37 (86.0 %)	6 (13.9 %)	
>12-15	23	19 (82.6 %)	4 (17.3 %)	
TOTAL	122	108 (88.5 %)	14 (11.4 %)	

**Figure 1:**
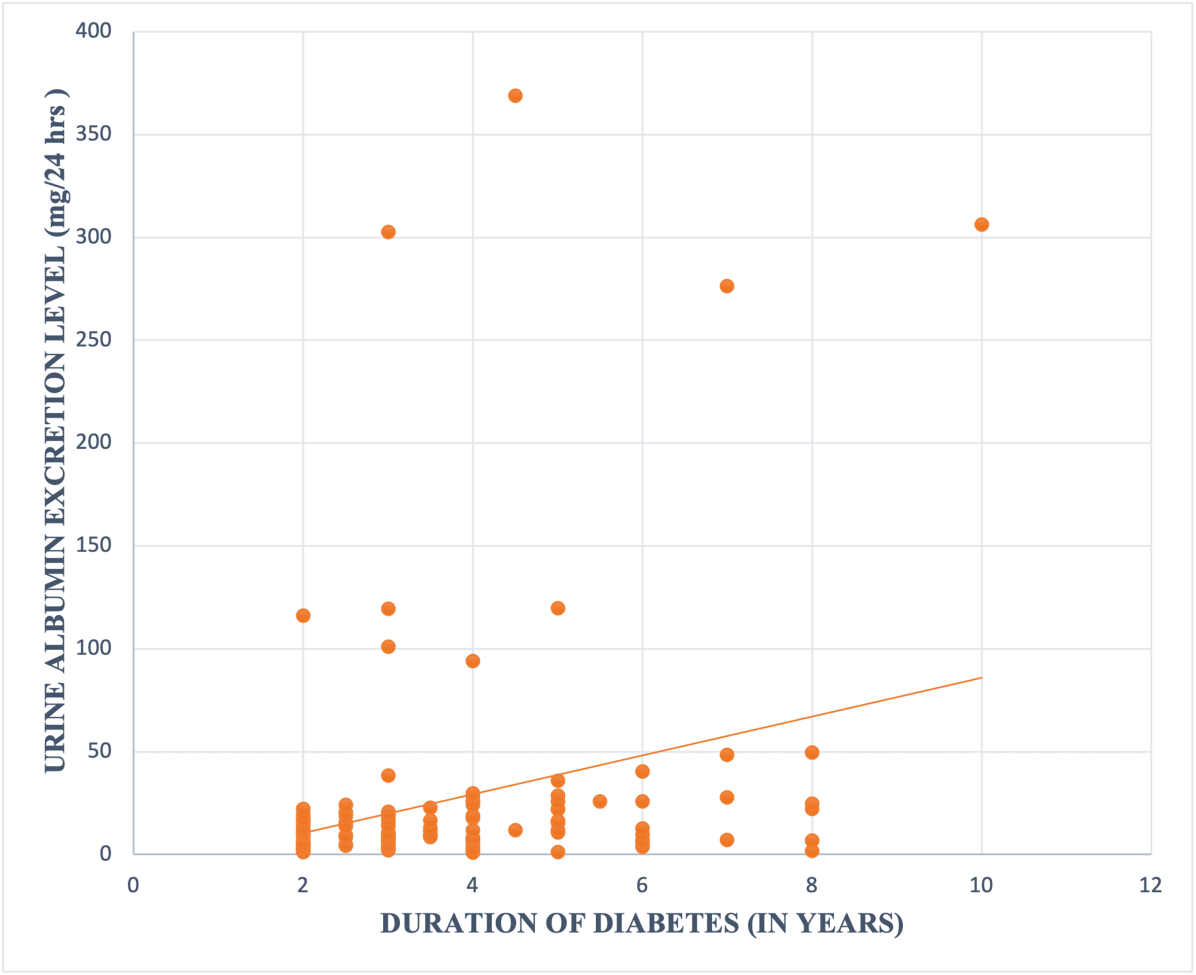
Correlation between Urine Albumin Excretion level and Duration of Diabetes (in years)

Significant linear correlation of UAE with duration of T1DM was observed (r 0.27, p value 0.002) (Figure 1).

T1DM cases with more than six years duration of disease had significantly higher proportion of cases with UAE >30 mg/24 hours (p value 0.023) (Table 5).

**Table 5:** Urine albumin excretion (>30 mg/24 hrs.) in relation to Duration of Diabetes (in years) *Chi-square test Chi-square score- 7.4944

Duration of T1DM in years	Number of patients (n, %)	Number of patients (n, %) with Urine albumin excretion (>30 mg/24 hrs.)	p-value*
2-4 years	90	6 (6.6 %)	.023
>4 -6 years	22	4 (18.1 %)	
>6 years	10	4 (40.0 %)	
Total	122	14 (11.4 %)	

**Figure 2:**
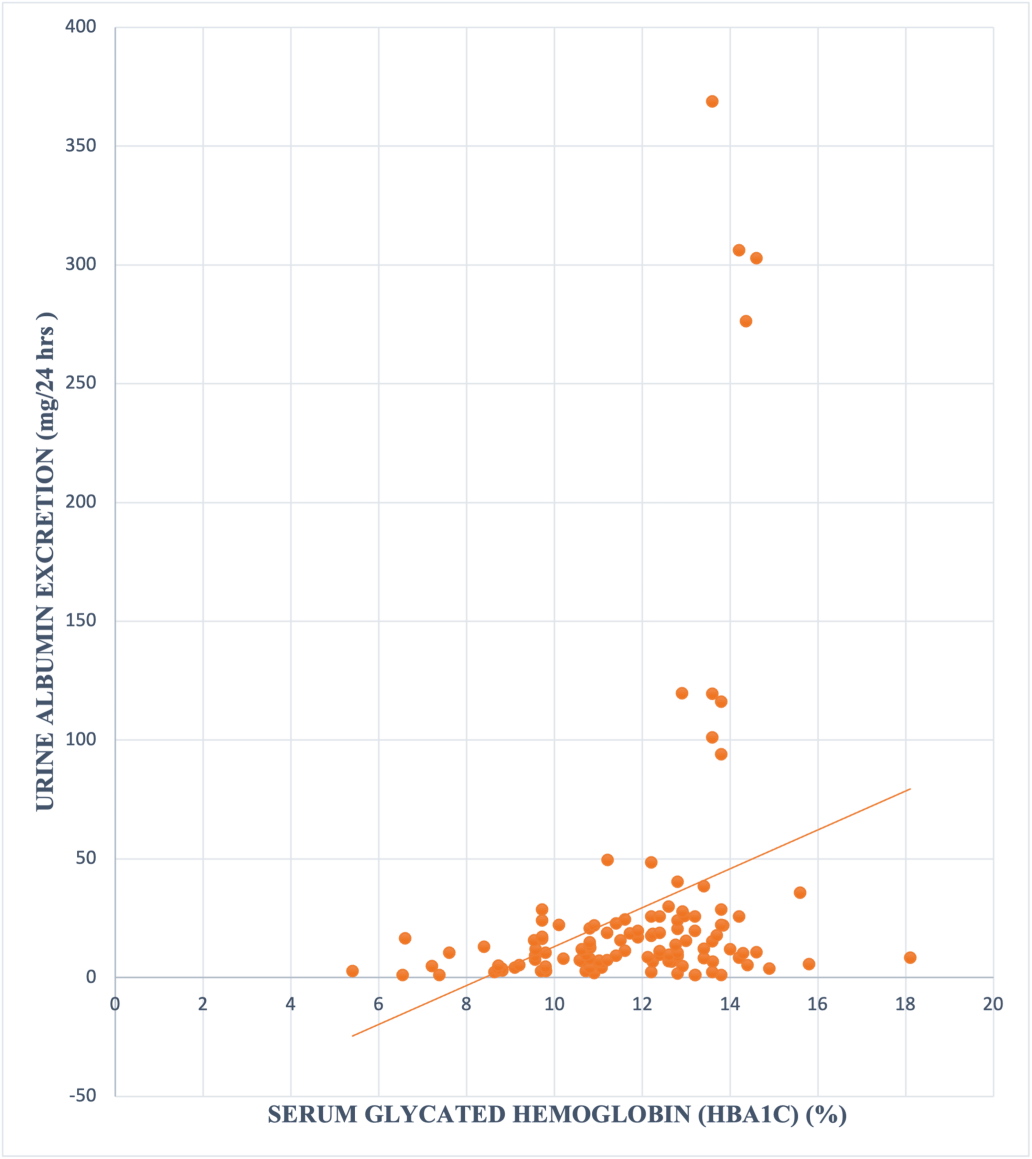
Graph showing correlation between serum glycated haemoglobin (HbA1c) and Urine Albumin excretion

Similarly, a significant linear correlation between rising HbA1c level and increasing UAE was also observed (r 0.30, p value 0.0007) (Figure 2).

Number of cases having UAE >30 mg/24 hours was significantly more in T1DM cases who has three or more episodes of DKA (p value 0.016) (Table 6).

**Table 6:** Relation of number of diabetic ketoacidosis (DKA) episodes with number of cases having urine albumin excretion (>30 mg/24 hrs.) *Chi-square test Chi-square score- 5.7798

Number of DKA episodes	Number of patients (n, %)	Number of patients without proteinuria (n, %)	Number of patients having Proteinuria (> 30 mg/24 hrs.) (n, %)	p-value*
<3	63	60 (95.2%)	3 (4.7%)	0.016
>=3	59	48 (81.3%)	11 (18.6%)	
Total	122	108 (88.5 %)	14 (11.4%)	

Analysis with one way ANOVA showing significant association between UAE and duration of T1DM (p value 0.002), HbA1c (p value 0.0003), total cholesterol level (p value <0.00001), triglycerides level (p value 0.018) and mean RRI (p value < .00001) while parameters high-density lipoprotein (HDL) and low-density lipoprotein (LDL) level were not significantly associated (Table 7).

**Table 7:** Various parameters in relation to urine albumin excretion *One-way ANOVA HbA1c: glycated hemoglobin, HDL: high-density lipoprotein, LDL: low-density lipoprotein, RRI: renal resistive index

Parameters	Normoalbuminuric (<=30 mg/24 hrs.)	Albuminuria (> 30 mg/24 hrs.)	F ratio*	P value*
Number of patients	108	14		
Mean Duration of Disease (Years)	3.5 ± 1.52	5.03 ± 2.3	9.95	0.002
Mean HbA1c (%)	11.45 ± 2.10	13.54 ± 1.06	13.39	0.0003
Mean Total Cholesterol (mg/dL)	167.95 ± 24.88	232.14 ± 50.59	61.53	<0.00001
Mean Triglycerides (mg/dL)	127.4 ± 23.46	143.7 ± 27.54	5.75	0.018
Mean HDL level (mg/dL)	45.8 ± 38.92	38.5 ± 7.93	0.49	0.48
Mean LDL Level (mg/dL)	94.8 ± 27.48	102.5 ± 31.5	0.9	0.33
Mean RRI	0.60 ± 0.04	0.73 ± 0.07	98.22	<0.00001

T1DM cases having UAE >30 mg/24 hours had significantly higher mean blood urea level (p value < .00001) and serum creatinine levels (p value < .00001) (Table 8).

**Table 8:** Mean Blood Urea level and Mean Serum Creatinine level in relation to Urine albumin excretion *ANOVA test

Parameters	Normoalbuminuric (<=30 mg/24 hrs.)	Albuminuria (> 30 mg/24 hrs.)	F ratio*	p-value*
Mean Blood Urea level (mg/dl)	28.9 ± 11.4	44.9 ± 29.7	30.58	<0.00001
Mean Serum Creatinine (mg/dL)	0.68 ± 0.18	0.97 ± 0.37	22.59	<0.00001

Female cases of T1DM had higher mean RRI (0.63 ± 0.07) as compared to males (0.61 ± 0.04) but the difference was not statistically significant (p-value 0.088) (Table 9).

**Table 9:** Mean Renal resistive index in relation to gender Unpaired T test T value 1.355

Gender	Mean Renal resistive index
Male	0.61 ± 0.04
Female	0.63 ± 0.07

**Figure 3:**
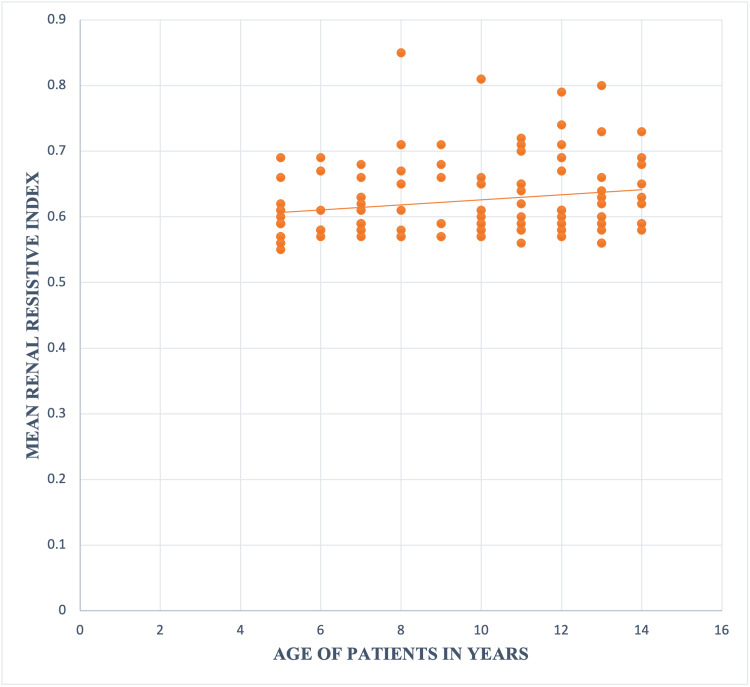
Correlation between Mean Renal resistive index and Age of Patients

Positive correlation of RRI with age of T1DM cases was observed (p value 0.043) (Figure 3).

Mean RRI was also significantly higher in cases with longer duration of diabetes (p value 0.005) (Table 10), higher HbA1c level (p value 0.022) (Table 11) and having three or more episodes of DKA (p value 0.0001) (Table 12).

**Table 10:** Mean renal resistive index in relation to duration of diabetes *ANOVA test T1DM: type 1 diabetes mellitus

Duration of T1DM in years	Mean Renal resistive index	F ratio*	p-value*
2-4 years	0.61 ± 0.05	5.547	0.005
>4 -6 years	0.63 ± 0.06		
>6 years	0.67 ± 0.07		

**Table 11:** Glycated haemoglobin (HbA1c) level (%) in relation to mean renal resistive index *ANOVA test F ratio: 3.326

HbA1C value (%)	Mean renal resistive index	p-value*
<= 8.5	0.58 ± 0.03	0.022
>8.5 – 10.5	0.59 ± 0.02	
>10.5-12.5	0.62 ± 0.04	
>12.5 – 14.5	0.64 ± 0.07	
>14.5	0.64 ± 0.08	

**Table 12:** Number of diabetic ketoacidosis (DKA) episodes in relation to mean renal resistive index *ANOVA test F ratio: 17.6665

Number of DKA episodes	Mean Renal resistive index	p-value*
<3	0.60 ± 0.04	0.0001
>=3	0.64 ± 0.06	

T1DM cases with UAE >30 mg/24 hours had significantly higher mean RRI (0.73 ± 0.07) as compared to patients without albuminuria (0.60 ± 0.04 ) (p value <0.00001) (Table 13).

**Table 13:** Urine albumin excretion in relation to mean renal resistive index *ANOVA test F ratio: 98.22

Urine Albumin excretion	Mean renal resistive index	p-value*
Cases with Normoalbuminuric (<=30 mg/24 hrs.)	0.60 ± 0.04	<0.00001
Cases with Albuminuria (>30 mg/24 hrs.)	0.73 ± 0.07	

**Figure 4:**
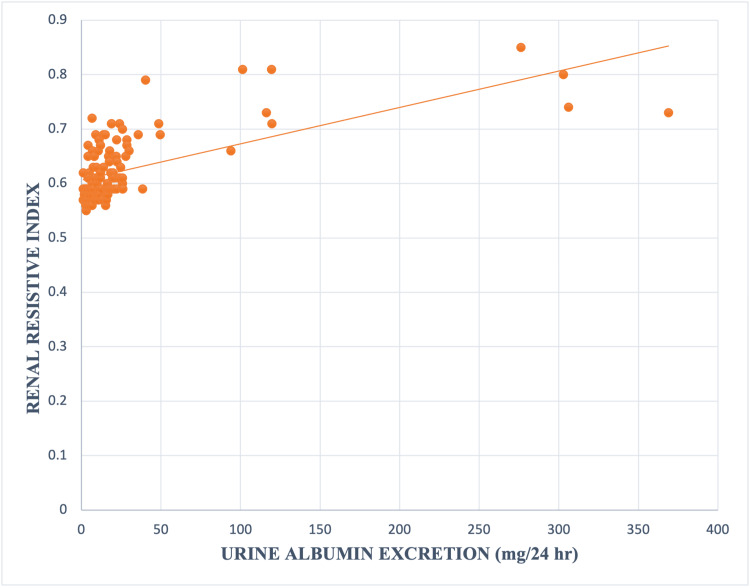
Relation between urine albumin excretion and renal resistive index

A strong significant linear correlation between UAE and RRI in T1DM cases was observed (r 0.63, p value <0.00001) (Figure 4).

**Figure 5:**
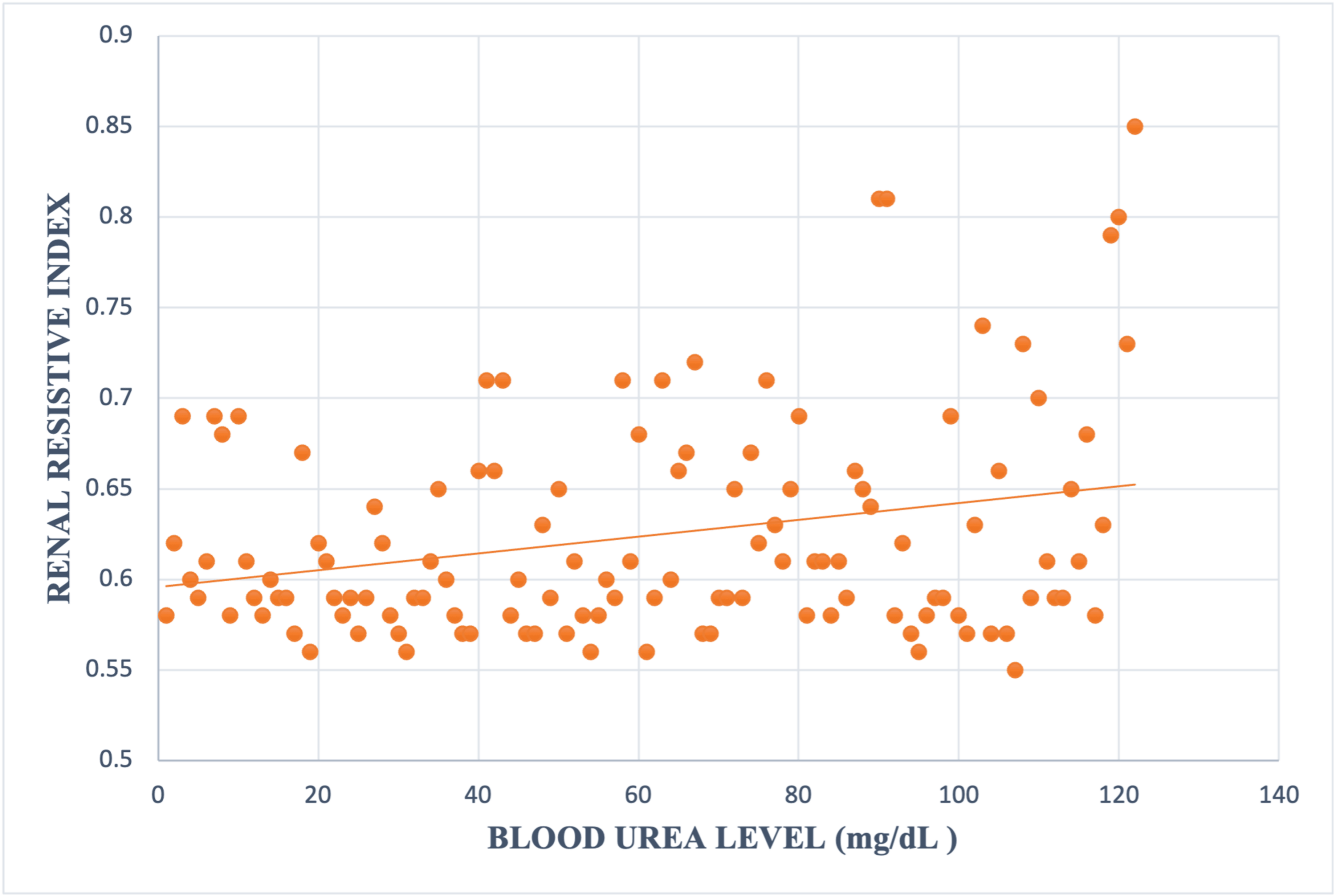
Relation between blood urea level and renal resistive index

Statistically significant linear correlation between RRI and blood urea level was observed (r 0.43, p value < 0.00001) (Figure 5).

**Figure 6:**
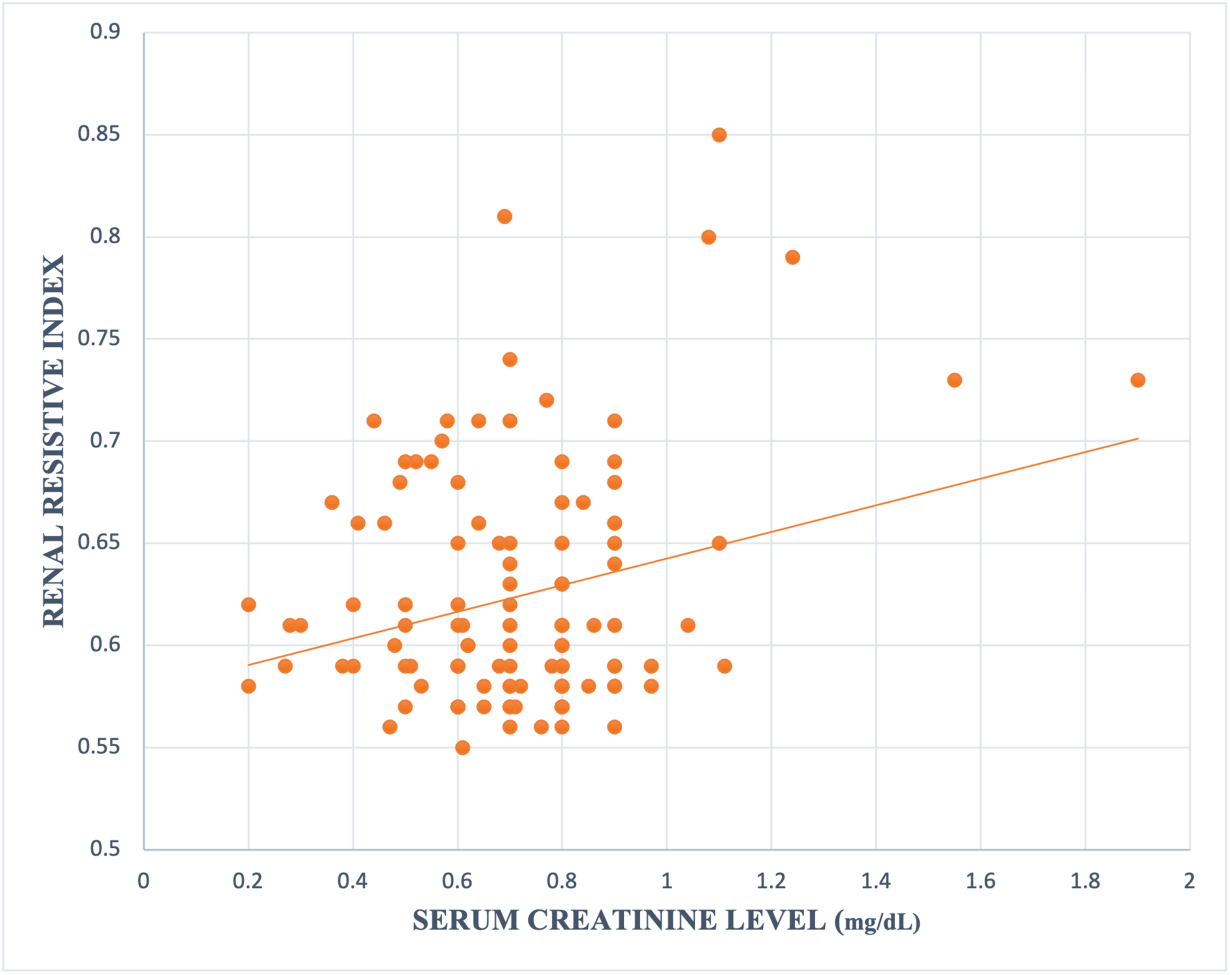
Relation between serum creatinine level and renal resistive index

A similar positive linear correlation was also observed between RRI and serum creatinine level (r 0.25, p value 0.005) (Figure 6).

Cut-off value of RRI for DN being >=0.7 [16], 15 (12.2%) cases of T1DM in our study had RRI >=0.7 indicative of DN (Table 14) and out of these 12 (80%) cases were females (Table 15).

**Table 14:** Distribution of cases according to mean renal resistive index

GROUP Name	Number of patients (n, %)
Group 1 (RI < 0.7)	107 (87.7 %)
Group 2 (RI >= 0.7)	15 (12.2 %)
Total	122 (100 %)

**Table 15:** Gender distribution of cases according to mean renal resistive index *Chi-square test Chi-square score- 5.83

Sex distribution	Group 1 (RI <0.7)	Group 2 (RI >=0.7)	P value*
Males	57 (53.2 %)	3 (20.0 %)	0.01
Females	50 (46.7 %)	12 (80 %)	
M: F ratio	1.14: 1	1: 4	
Total	107 (100 %)	15 (100 %)	

The majority of cases (103) with RRI <0.7 didn’t have albuminuria, while the majority of cases (10) with RRI>=0.7 had UAE >30 mg/24 hours. This difference was statistically significant (p value < 0.00001) (Table 16).

**Table 16:** Urine albumin excretion in renal resistive index (RRI) Group 1 and Group 2 *Chi-square test Chi-square score- 51.2833

Group	Number of patients (n, %)	Number of cases (n, %) with Urine albumin excretion (<=30 mg/24 hrs.)	Number of patients (n) with Urine albumin excretion (>30 mg/24 hrs.)	P value*
Group 1 (RI <0.7)	107	103 (96.2%)	4 (3.7%)	< .00001
Group 2 (RI >= 0.7)	15	5 (33.3 %)	10 (66.6 %)	
Total	122	108 (88.5%)	14 (11.4 %)	

Mean blood urea and mean serum creatinine levels were also significantly higher in T1DM cases with RRI >=0.7 i.e., 51.16 ± 32.03 (p value < 0.00001) and 0.9 ± 0.40 (p value 0.0007), respectively (Table 17).

**Table 17:** Mean blood urea and mean serum creatinine in renal resistive index (RRI) Group 1 and Group 2 *ANOVA test

Parameters	Group 1 (RI <0.7)	Group 2 (RI >= 0.7)	F ratio*	p-value*
Mean Blood Urea level (mg/dl)	28.94 ± 11.66	51.16 ± 32.03	27.06	<0.00001
Mean Serum Creatinine (mg/dL)	0.69±0.18	0.9±0.40	11.84	0.0007

On ROC curve analysis in our study the best discriminative point of differentiation of RRI between T1DM cases having albuminuria >30 mg/24 hours or not was found to be >=0.69 (Table 18). At this cut-off point the sensitivity and specificity were 85.7% and 92.6% respectively. The PPV and NPV was 60.0% and 98.0% respectively.

**Table 18:** Cross tabulation of renal resistive index (RRI) for maximum sensitivity and specificity

Cut-off Mean RI	Sensitivity	Specificity	Youden	PPV	NPV
≥ 0.0	100.0%	0.0%	0.0	11.5%	0.0%
≥ 0.55	100.0%	0.0%	0.0	11.5%	0.0%
≥ 0.57	100.0%	5.6%	0.056	12.1%	100.0%
≥ 0.59	100.0%	32.4%	0.324	16.1%	100.0%
≥ 0.61	92.9%	56.5%	0.493	21.7%	98.4%
≥ 0.63	92.9%	72.2%	0.651	30.2%	98.7%
≥ 0.65	92.9%	77.8%	0.706	35.1%	98.8%
≥ 0.66	92.9%	83.3%	0.762	41.9%	98.9%
≥ 0.67	85.7%	87.0%	0.728	46.2%	97.9%
≥ 0.68	85.7%	89.8%	0.755	52.2%	98.0%
≥ 0.69	85.7%	92.6%	0.783	60.0%	98.0%
≥ 0.7	71.4%	95.4%	0.668	66.7%	96.3%
≥ 0.71	71.4%	96.3%	0.677	71.4%	96.3%
≥ 0.74	42.9%	100.0%	0.429	100.0%	93.1%
≥ 0.79	35.7%	100.0%	0.357	100.0%	92.3%
≥ 0.85	7.1%	100.0%	0.071	100.0%	89.3%

When RRI >=0.69 is taken as the cut-off point indicative of DN, the prevalence is 16.3% in our study, while prevalence according to UAE (>30 mg/24 hours) is 11.4% (Table 19).

**Table 19:** Distribution of cases according to best cut-off value of mean renal resistive index (RRI) (>=0.69)

RRI value	Number of patients (n, %)
RI < 0.69	102 (83.6 %)
RI >= 0.69	20 (16.3 %)
Total	122 (100 %)

## Discussion

According to a study, 30 to 40% of patients with T1DM develop DN in the later course of the disease [17]. Not much literature is available about the role and the predictive value of RRI in patients with DN. Only a few studies have been done in the past describing the utilization of renal Doppler for the evaluation of intra-renal hemodynamic abnormalities in DN, and most of these studies have been conducted in adults with Type 1 or Type 2 DM. Thus, in the present study evaluation of mean RRI by renal colour Doppler with some routine investigations was done in children with T1DM to evaluate its role in early detection of DN.

The prevalence of albuminuria (>30 mg/24 hours) in our study was 11.4% (14 out of 122 cases), whereas the prevalence of children with T1DM having mean RRI >=0.7 was 12.29% (15 out of 122 cases). This low prevalence of patients suspected with DN can be explained by the observation that the maximum number of cases in our study had the disease for two to four years (73.7 %) and the incidence of microalbuminuria usually occurs after 15 years of onset of diabetes [18]. In our study, maximum number of cases were in the five to nine years age group (45.9% of cases) indicating that most of the children in this study had onset of T1DM before reaching adolescent age and by previous study it has been proved that patients who have T1DM from early childhood seem to have a slightly delayed onset of albuminuria during the initial 10 to 15 years after diagnosis of diabetes [19]. Even in diabetics of long duration, albuminuria onset is rare before the child reaches adolescent age [20].

Poor metabolic control is widely acknowledged to increase the risk of both microvascular and macrovascular complications in patients with T1DM. Although threshold values may vary across studies, HbA1c levels above 7.5% are frequently used as a practical indicator of suboptimal glycemic control [2,3,12]. In our study, the mean HbA1c level among patients was 11.69 ± 2.1%, reflecting poor glycemic control. This suboptimal control is primarily attributed to inadequate blood glucose monitoring at home, improper insulin storage, poor dietary habits, and low socioeconomic status. Similarly, a study involving 25 children with T1DM and 20 age-matched healthy controls reported significantly higher HbA1c levels in the diabetic group (8.9 ± 0.8%) compared to the control group (5.2 ± 0.57%) [21].

Our study demonstrated that diabetic patients with elevated serum HbA1c levels showed a significant positive correlation with UAE (p = 0.0007) and an increase in mean RRI (p < 0.00001). These findings are consistent with those of a previous study conducted on 100 children with T1DM, which reported that patients with microalbuminuria had a significant correlation with elevated HbA1c levels (p<0.05). Furthermore, HbA1c levels greater than 7.5% were significantly associated with increased UAE and RRI (p < 0.01) [22].

In our study, a statistically significant correlation was observed between longer duration of T1DM and both increased UAE levels (p = 0.002) and elevated mean RRI (p = 0.005). This observation aligns with the findings of a previous study, which identified the duration of diabetes as one of the most important risk factors for the development of DN, surpassing other factors such as age, sex, or type of diabetes [23].

Kidney biopsy remains the gold standard for distinguishing between DKD and non-diabetic kidney disease (NDKD) in terms of clinical diagnosis and treatment; however, it is not always feasible in clinical practice due to its invasive nature [24]. In our study, renal Doppler ultrasound was utilized as a non-invasive and cost-effective alternative. This technique provides valuable insights into renal hemodynamics, with peak systolic velocity primarily reflecting the degree of renal vascular filling and blood supply, while end-diastolic velocity indicates renal blood perfusion. The RRI serves as a key parameter, mainly representing vascular bed resistance [25].

A study involving 469 patients with type 2 diabetes mellitus found that RRI values were significantly higher in the DKD group compared to the NDKD group, and that an RRI-based differential model demonstrated good sensitivity and specificity for distinguishing between the two conditions [26]. Additionally, Doppler sonography has been shown to detect early renal hemodynamic changes in children with diabetes who do not yet exhibit clinical signs of renal dysfunction, potentially identifying a preclinical stage of DN [27]. A 2019 study further supported this, showing that RRI increases more rapidly in diabetic patients than in non-diabetics, independent of albuminuria status. Moreover, RRI values were notably higher in diabetic patients with chronic kidney disease compared to non-diabetic counterparts [28]. It is important to note, however, that there is currently no universally accepted standard for normal average RRI values.

A study done in diabetic subjects showed that the RRI correlated well with renal function and its value >=0.70 can be considered as pathologic values [16]. In their study, RRI value >= 0.70 were observed in 15% cases in the asymptomatic group and in 87% in the group with advanced nephropathy. Based on these findings, our study considered an RRI value of ≥0.70 as the threshold indicative of increased renal vasoconstriction.

In our study, a statistically significant increase in mean RRI was observed with longer duration of T1DM (p value 0.005) and higher HbA1c levels (p value 0.022). These findings are consistent with those of a previous study, which reported a strong positive correlation between elevated RRI and both HbA1c levels and diabetes duration [29]. That study also highlighted the importance of maintaining good glycemic control to prevent or delay the onset of renal complications.

Our study found that children with T1DM and albuminuria (>30 mg/24 hours) had a significantly higher mean RRI of 0.73 ± 0.07, compared to non-albuminuric patients who had a mean RRI of 0.60 ± 0.04 (p < 0.00001). These findings are consistent with previous research. One study reported a significant increase in mean RRI among children with T1DM compared to age-matched healthy controls (0.64 ± 0.55 vs. 0.58 ± 0.28) [21]. Similarly, another study observed the highest mean RRI (0.59) in microalbuminuric patients, whereas the control group had a mean RRI of 0.54 [22].

Based on the results of our study, patients with a mean RRI ≥0.7 had a significantly higher prevalence of elevated UAE (>30 mg/24 hours) (p < 0.00001). These findings are consistent with those of a previous study, which reported significantly higher mean RRI values in patients with elevated UAE compared to those with normal albumin excretion and healthy controls [30].

Furthermore, in our study, mean blood urea and serum creatinine levels were significantly higher in the group with RRI ≥0.7 compared to Group 1 (p < 0.00001 and p = 0.0007, respectively). These results are in line with the findings of a previous study, which also demonstrated significantly higher mean RRI values in patients with elevated serum creatinine levels. Additionally, multiple regression analysis in that study revealed that RRI values in diabetic patients were significantly influenced by creatinine clearance, age, and duration of diabetes [30].

These results indicate that patients with T1DM and nephropathy exhibit intrarenal hemodynamic abnormalities, which are reflected by changes in the RRI of the segmental renal arteries. Notably, an elevated RRI may precede the onset of microalbuminuria, suggesting that it could serve as an early and sensitive marker for the early detection of DN.

However, this study has several limitations. It was a single-center, hospital-based study with a relatively small sample size, which may limit the generalizability of the findings to the wider population of diabetic patients. Furthermore, the short duration of the study restricted the ability to evaluate long-term outcomes and hindered a comprehensive assessment of potential predictors of diabetic nephropathy.

## Conclusions

In our study, the prevalence of DN, defined as UAE >30 mg/24 hours, among children with T1DM aged five to 15 years was 11.4% (14 cases). Significant risk factors associated with DN included female sex, older age, longer duration of diabetes, elevated total cholesterol and triglyceride levels. Poor glycemic control also emerged as a key risk factor, as evidenced by higher mean HbA1c levels and a greater number of DKA episodes among those with DN.

An elevated renal resistive index (RRI ≥0.7), suggestive of DN, was observed in 12.2% (15 cases) of the T1DM cohort. ROC curve analysis identified an RRI cut-off value of ≥0.69 as the most predictive threshold for DN in our study. At this cut-off, the prevalence of DN was 16.3% (20 cases), which is higher than that detected using the UAE criterion (11.4%), indicating that RRI may be a more sensitive and earlier marker for detecting DN in T1DM patients.

Furthermore, T1DM cases with both UAE >30 mg/24 hours and RRI ≥0.7 exhibited significantly higher mean blood urea and serum creatinine levels, further supporting renal involvement. The significant correlation between increased RRI and elevated UAE underscores the utility of RRI as a reliable, non-invasive diagnostic tool for the early detection of diabetic nephropathy in children with T1DM.
